# Long-Term Simulated Atmospheric Nitrogen Deposition Alters Leaf and Fine Root Decomposition

**DOI:** 10.1007/s10021-017-0130-3

**Published:** 2018

**Authors:** Mengxue Xia, Alan F. Talhelm, Kurt S. Pregitzer

**Affiliations:** 1 College of Natural Resources, University of Idaho, Moscow, Idaho 83844, USA; 2 Oak Ridge Institute for Science and Education, National Center for Environmental Assessment, US Environmental Protection Agency, Research Triangle Park, North Carolina 27709, USA

**Keywords:** fine roots, initial litter chemistry, litter decomposition, leaf litter, Michigan Gradient Study, nitrogen deposition, soil organic carbon, sugar maple

## Abstract

Atmospheric nitrogen deposition increases forest carbon sequestration across broad parts of the Northern Hemisphere. Slower organic matter decomposition and greater soil carbon accumulation could contribute to this increase in carbon sequestration. We investigated the effects of chronic simulated nitrogen deposition on leaf litter and fine root decomposition at four sugar maple (*Acer saccharum*)- dominated northern hardwood forests. At these sites, we previously observed that nitrogen additions increased soil organic carbon and altered litter chemistry. We conducted a 3-year decomposition study with litter bags. Litter production of leaves and fine roots were combined with decomposition dynamics to estimate how fine roots and leaf litter contribute to soil organic carbon. We found that nitrogen additions marginally stimulated early-stage decomposition of leaf litter, an effect associated with previously documented changes in litter chemistry. In contrast, nitrogen additions inhibited the later stages of fine root decomposition, which is consistent with observed decreases in lignin-degrading enzyme activities with nitrogen additions at these sites. At the ecosystem scale, slower fine root decomposition led to additional root mass retention (g m^−2^), and this greater retention of root residues was estimated to explain 5–51% of previously documented carbon accumulation in the surface soil due to nitrogen additions. Our results demonstrated that simulated nitrogen deposition created contrasting effects on the decomposition of leaf litter and fine roots. Although previous nitrogen deposition studies have focused on leaf litter, this work suggests that slower fine root decomposition is a major driver of soil organic carbon accumulation under elevated nitrogen deposition.

## Introduction

Human activities currently convert more atmospheric nitrogen (N) gas to biologically active forms of N than all natural processes combined (Gruber and Galloway 2008). A large portion of the reactive N created by human activity is added to terrestrial ecosystems via atmospheric N deposition, substantially increasing reactive N inputs across wide areas of Europe, North America, and Asia (Gruber and Galloway 2008; Liu and others 2013). Investigations across boreal and temperate forests in Western Europe and North America showed that atmospheric N deposition is a major driver of forest carbon (C) accumulation (Sutton and others 2008). Because N availability limits plant productivity in most terrestrial ecosystems (LeBauer and Treseder 2008), greater tree growth due to higher N availability appears to contribute to the N deposition-induced C sink. Forest soil also represents a large C pool in boreal/temperate biomes (Pregitzer and Euskirchen 2004) that could be sensitive to anthropogenic N deposition (for example, Yue and others 2016). Indeed, several chronic experimental N deposition studies in northern temperate forests have observed that long-term N additions increased soil organic C storage (Franklin and others 2003; Hyvonen and others 2008; Pregitzer and others 2008; Frey and others 2014). Given these observations, knowledge of how and why soil C pools respond to added N is crucial for understanding the extent to which terrestrial C cycling is altered by N deposition.

At four northern temperate forests in the north-central USA, simulated N deposition has been applied to replicated plots as part of the Michigan Gradient Study (MGS) since 1994. One of the major observations from this experiment is that chronic N additions increased the C pool in the soil organic horizons and surface mineral soil by about 26% (Pregitzer and others 2008). This increase in soil C occurred without an increase in litter input, providing evidence for slower decomposition of organic matter under simulated N deposition (Zak and others 2008). The mechanisms underlying this slower decomposition of organic matter with elevated N deposition are not fully understood.

Elevated N deposition could both increase soil N availability and alter initial litter chemistry (chemistry prior to decomposition); each of these changes may affect litter decomposition. Variation in litter chemistry has been linked with differences in decomposition rates (Taylor and others 1989; Currie and others 2010). Our previous work at MGS sites reported that simulated N deposition caused changes in the initial chemistry of leaf litter, including increasing N concentration, decreasing the concentration of condensed tannins, the acid-insoluble fractions (AIF), and the AIF/N ratios (Xia and others 2015). These chemical changes have been associated with faster decomposition (Taylor and others 1989; Currie and others 2010; Hättenschwiler and Jørgensen 2010). In contrast, initial chemical properties in fine roots were relatively unresponsive to simulated N deposition. Thus, it is possible that simulated N deposition has altered the initial chemistry of leaf litter so that it decomposes more quickly than litter produced under ambient conditions, but there is no evidence of a similar effect on fine roots.

Soil/external N availability has also been observed to affect litter decomposition. Knorr and others (2005) conducted a meta-analysis on the effects of N additions on leaf litter decomposition. The results indicated that added N inhibited decomposition when ambient N deposition was relatively high (5–10 kg N ha^−1^ y^−1^), when litter was lignin-rich, and when experiments lasted more than 2 years; by contrast, adding N accelerated decomposition when ambient N deposition was low, among lignin-poor litter substrates, and in short-term studies. Individual litter decomposition studies lasting more than 4 years found that, regardless of initial lignin levels, externally supplied N stimulated initial decomposition, but eventually inhibited the later stages of decomposition that were likely dominated by lignin degradation (Hobbie and others 2012; Sun and others 2016). Fog (1988) proposed that exogenous N inhibits decomposition by forming recalcitrant complexes with lignin degradation intermediate products or by suppressing microbial activity. Consistent with the latter hypothesis, recent microbial analyses showed that experimental N enrichment generally inhibits microbial degradation of more recalcitrant carbon fractions such as lignin: N additions were observed to reduce microbial respiration and biomass (Frey and others 2014; Ramirez and others 2012), promote copiotrophic bacteria groups that preferentially utilize labile C over more recalcitrant compounds (Ramirez and others 2012), decrease the activity of lignin-degrading extracellular enzymes (DeForest and others 2004; Sun and others 2016), suppress the expression of the gene encoding for the lignin-degrading laccase (Edward and others 2011), and favor bacterial metabolisms that are less powerful to oxidize lignin relative to fungal pathways (Freedman and others 2016). These changes in microbial function suggest that elevated soil N availability could be slowing litter decomposition at MGS sites by suppressing microbial metabolism of lignin.

Studies on the effects of experimental N deposition on litter decomposition have focused on leaf litter, while fine roots also represent large quantities of litter fluxes (Xia and others 2015). Compared to leaf litter, fine roots generally contain more lignin (Rasse and others 2005; Xia and others 2015). The above evidence suggesting that N additions suppress lignin-degrading metabolism and the observations that added N tends to suppress the decomposition of litters with greater lignin contents (Carreiro and others 2000; Sinsabaugh and others 2002; Knorr and others 2005) both point to the idea that N additions could impose stronger inhibitory effects on decomposition of fine roots than leaf litter. Because litter fluxes of fine roots and leaves are comparable in size at MGS sites and fine roots contain 2.9-fold more acid-insoluble fraction (AIF, conventionally referred to as lignin, but also contains other recalcitrant substrates such as cutin and condensed tannin, Preston and others 2009), it is possible that fine roots are the major driver of slower organic matter turnover with simulated N deposition at MGS sites.

The objective of this study was to investigate the effects of simulated N deposition on leaf litter and fine root decomposition at MGS sites, which are heavily dominated by sugar maple (*Acer saccharum* Marsh.). We hypothesized that both altered substrate biochemistry and elevated soil N availability shape the overall effects of simulated N deposition on litter decomposition. However, their roles are different for leaf litter and fine roots. Specifically, we hypothesized that simulated N deposition stimulated the decomposition of leaf litter because long-term experimental N additions have caused changes in initial litter chemistry that made leaves easier to degrade. In contrast, we hypothesized that simulated N deposition would inhibit decomposition of fine roots because external N enrichment suppresses microbial metabolism of lignin and lignin is comparatively high in fine roots. We tested the effects of simulated N deposition on leaf litter and fine root decomposition by a 3-year decomposition study across four MGS sites. We investigated the relationship between initial substrate chemistry and decomposition rates for leaf litter and fine roots. Further, fine roots were decomposed reciprocally as an attempt to disentangle the effects of initial substrate and external N availability on litter decomposition. By combining annual litter inputs with estimates of litter decomposition dynamics, we were able to make ecosystem-scale estimates of how simulated N deposition affected the mass retention per unit area of fine roots and leaf litter in the soil.

## Materials and methods

### Site Description

The four MGS sites span a 500-km climate and ambient N deposition gradient in Michigan, USA, which covers the north–south distribution of the northern hardwood forest biome in the Great Lakes region (Table 1). These sites are dominated by sugar maple, similar in stand composition and overstory age. The soil O_e/a_ horizons are permeated by a dense growth of sugar maple roots at all sites. Soils are sandy (Kalkaska series, Typic Haplorthods), with pH values ranging from 4.4 to 4.7 in the top 10-cm mineral soil. Within each site, six 30-m × 30-m plots were established in 1994. Each plot was surrounded on all sides by a 10-m wide buffer zone that received the same treatment as the main plot. The three treatment plots at each site received ambient N deposition plus an experimental addition of 3 g N m^−2^ y^−1^ as NaNO_3_ in six equal increments across the growing season, a rate similar to those occur in some areas of Europe and China (Holland and others 2005; Liu and others 2013). Until recently, NO_3_^−^ was the dominant form of wet N deposition in this region (Talhelm and others 2012).

### Litter Sampling and Chemical Analysis

Sugar maple leaf litter was collected in the autumn of 2010 following the protocol of Pregitzer and others (2008) from litter traps located in each plot. Fine roots were excavated from the top 10-cm soil, including the O_e+a_ horizons, at six to ten random spots within the buffer zone surrounding each plot in October 2010 (autumn) and May 2011 (spring). After being removed from soil, roots were identified to the genus *Acer* by morphological characteristics, washed and flash-frozen in liquid N_2_ before being transported to the University of Idaho on dry ice. We collected fresh roots instead of dead root litter because it was impractical to identify large enough quantities of recently senesced fine roots for this study. Nutrient resorption and other biochemical changes during senescence are less understood in roots than in leaves (Comas and others 2000). Compared to the drastic changes in leaf biochemistry during senescence, previous root studies observed either no or considerably less nutrient resorption (Gordon and Jackson 2000). We excluded white and turgid roots to minimize the difference between the roots we sampled and root necromass. Because sugar maple leaf litter represented about 77% of leaf litter flux at these sites, and the genus *Acer* contributed up to 90% of overstory basal area and 83% of woody seedling groundcover stems (Talhelm and others 2013), the leaf litter and fine roots we sampled are broadly representative of the litter produced in these forests.

For initial chemistry analysis and decomposition study, we isolated first to third branch orders of fine roots following the procedures in Pregitzer and others (2002). Fine roots of these distal orders represent the most short-lived and metabolically active portions of root systems (Guo and others 2008; Xia and others 2010). Although these distal roots do not necessarily represent a large portion of total root standing biomass, they are the major driver of root mass and N turnover due to their shorter lifespan (McCormack and others 2015). In contrast, traditionally defined fine roots (< 2 mm in diameter) often contain a significant portion of roots that have undergone secondary thickening and have a much longer lifespan (Xia and others 2010; McCormack and others 2015).

All samples were oven-dried (50°C) for two days. Approximately 2 g DW of leaf litter and fine roots from each plot was used to determine initial litter chemical characteristics, including concentrations of C and N, nonstructural carbohydrates, soluble phenolics, condensed tannins, soluble proteins, total lipids, acid-insoluble fraction, acid-soluble fraction, and ash. The details of chemical analysis were shown in Xia and others (2015).

### Decomposition Study

About 1 g DW of leaf litter or fine roots was sealed into 20 cm × 20 cm polyester litterbags. The mesh sizes of litterbags were 20 μm on the bottom and 300 μm on the top. The bottom mesh allowed fungal hyphae to penetrate while minimizing physical loss of plant debris (Hobbie 2005); the top mesh permitted entry of micro-fauna, but likely excluded entry of larger soil fauna (Bradford and others 2002). Thus, this study focused on the decomposition driven mostly by microorganisms, and caution remains as to the potential effects of soil animals.

Litterbags were returned to MGS sites for decomposition starting in July 2011. Leaf litter was deployed in situ to represent native leaf litter fall. Because the litter traps did not yield enough leaf litter mass from some plots for this study, leaf litter collected from the three plots receiving the same treatment at each site was homogenized before being sealed into litterbags and deployed to those three plots. Leaf litter bags were placed flat on the top of the forest floor, that is, O horizon surface. Additional leaf litter bags were placed at the interface between organic horizons and mineral soil, that is, O/A interface, to rule out environmental effects in the differences between leaf litter and fine root decomposition. For fine roots, we collected enough initial material for a reciprocal deployment between ambient and N-added plots so that we could disentangle the effects of initial substrate and externally supplied N. Specifically, besides being deployed in the original plots, fine roots were also placed in the plots of the alternate treatment at the same site. All root bags were placed at O/A interface. Because root mortality occurs relatively evenly throughout growing season at MGS sites (Burton and others 2000), both autumn and spring roots were decomposed to better characterize fine root decomposition within a plot. Autumn and spring roots of a treatment type were placed in separate litterbags in a plot; the final root mass remaining of that plot was calculated as the mean of these two litterbags. Taken together, we had six types of litterbags (two leaf litters and four fine roots) in each plot at each site, with each type in a plot having 18 replicate bags. We deployed 2592 litterbags in total.

Three replicate bags of each type in a plot were harvested each time after periods of 1, 3 months, 1, 2, and 3 years. Harvested bags were flash-frozen in liquid N_2_ and transported to the University of Idaho on dry ice. We removed sample material from the litterbags and cleaned them of soil, new root growth, and animal necromass. Decomposing samples were then freeze-dried and weighed. Mass loss in a litterbag was calculated as the difference between the dry mass of initial and harvested substrates on the ash-free basis (500°C for 4 h). Plots are the experimental units in the data analysis, so we averaged the mass remaining of three replicate bags from one plot.

### Data Analysis

Data analyses were conducted using SAS software (version 9.3; SAS Institute Inc., Cary, NC). We evaluated decomposition dynamics using three commonly used decomposition models (Wieder and Lang 1982; Harmon and others 2009): a single-exponential decay model, *M*_*t*_ = *e*^−*k_s_t*^; a double-exponential decay model, *M_t_* = *Ae*^−*k*_1_*t*^ + (1 − *A*)*e*^−*k*_2_*t*^; and an asymptotic model, *M_t_* = (1 − *S*)*e*^−*k*_a_*t*^ + *S*. Here, *M_t_* is the percentage of mass remaining at time *t* (year) and *k* is the decomposition rate of a certain substrate fraction. In single-exponential models, the whole substrate decomposes at the rate *k*_*s*_. In double-exponential models, *A* is the kinetically defined active fraction of the substrate that decomposes at a higher rate *k*_1_, whereas (1 − *A*) is the fraction with a lower *k*_2_. In asymptotic models, *S* represents a stable fraction with a decomposition rate of zero, whereas (1 − *S*) decomposes at a rate *k*_a_. These models make different biological assumptions about decomposition process (Wieder and Lang 1982). Single-exponential models assume that substrates decompose at a constant decomposition rate. Both double-exponential and asymptotic models assume that substrates decompose as two pools, but asymptotic models assume a completely stable slow pool. Model performance was assessed with both adjusted *R*^2^ and the Akaike Information Criterion corrected for small sample size (AICc), where the AICc differences > 7 indicate significant difference in model performance (Burnham and others 2011). Due to the small sample size (that is, time points) in one plot for a litter type, we pooled together time points of three ambient or N-amended plots at each site to assess model performance (*n* = 18). When the overall “best-fit” model was determined, we fitted each plot to this model to estimate individual decomposition parameters for further analysis (*n* = 6). The active pool (*A*) in double-exponential models is considered an initial litter trait (Wieder and Lang 1982); thus, decomposition patterns of leaf litter or fine roots collected from the same plot were constrained to have the same *A* value (Table 2).

The effects of simulated N deposition on leaf litter and fine root decomposition were evaluated using model-fitted decomposition rates (y^−1^), proportions of mass remaining (%), and quantity of mass remaining (g m^−2^). Because the effects of N additions may be more manifest in the later stage of decomposition, we extrapolated the mass remaining beyond the study period using the overall “best-fit” model at the plot level. Aber and others (1990) showed that extrapolation of exponential decomposition models was valid until litter decomposition shifted to a more stable phase when the majority of the initial mass had been lost. Previous long-term decomposition studies in similar temperate forests showed that decomposing leaf litter seemed to be more stable after 6–7 years, when less than 20% of initial mass remained (Adair and others 2008; Harmon and others 2009). Therefore, we extrapolated decomposition to 6 years. We note that this extrapolation and statistical inferences drawn from this extrapolation should be interpreted with caution. We estimated the quantity of mass remaining of an annual litter cohort in each plot by multiplying the annual litter inputs with the proportions of mass remaining. Annual litter inputs for leaves and fine roots were estimated at the plot level (*see* Xia and others 2015).

A two-way ANOVA was used to test whether each of the decomposition metrics was different among sites (*df* = 3) and N treatment (*df* = 1) for leaf litter and fine roots decomposed in situ. Alternatively, we conducted an ANCOVA analysis on decomposition metrics with site as the main effect and each of the initial chemical traits as a covariate tested in a separate ANCOVA analysis. Our previous work has shown that simulated N deposition affected initial litter biochemistry (Xia and others 2015). Here, the initial chemical traits were used as variables to relate chemical changes due to N additions to the variation in decomposition metrics. A common slope model of all sites was used because initial ANCOVA failed to reject the hypothesis that slopes were equal across sites (Littell and others 2006). Because fine roots were decomposed reciprocally between ambient and N added conditions, we further analyzed decomposition metrics of fine roots with factorial arrangements of substrate source and external N availability. We tested whether substrate source (litter collected from N-amended vs. ambient, *df* = 1), external N availability (litterbags deployed in N-amended vs. ambient, *df* = 1), and study sites (*df* = 3) affect fine root decomposition metrics with a mixed linear model (Proc mixed, Littell and others 2006) in a split-plot design. Site, external N availability and their interactions (*df* = 3) were tested on whole-plot experimental units (that is, plots in site × external N treatment), whereas substrate source is the within-plot factor. Data were log-transformed when needed to improve homogeneity of variance and normality before being analyzed.

## Results

**Figure 1.**
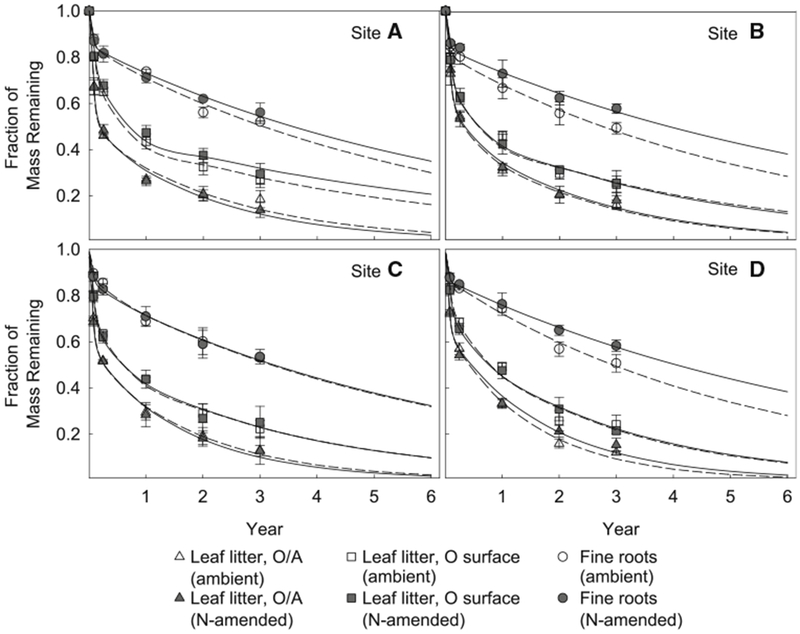
Decomposition patterns of leaf litter and fine roots across four northern hardwood forests. Each data point is the mean with SD of three ambient or N-amended plots at each site (*n* = 3). *Dash lines* are the double-exponential model-predicted decomposition patterns in ambient plots, whereas *solid lines* are those under simulated N deposition.

The goals of this study were to understand decomposition of leaf litter and fine roots at four northern hardwood forests and how these patterns were altered by chronic simulated N deposition. Averaged across sites, fine roots had the highest proportion of mass remaining after 3 years (51.29–56.40%), followed by leaf litter deployed on O horizon surface (24.60–25.41%), and leaf litter at O/A interface (14.96–15.06%; Figure 1; Table 3). All three-exponential models exhibited strong fits (*R*^2^ > 70% in most cases), but double-exponential models generally exhibited the best balance between accuracy and parsimony (*R*^2^ > 90%, lowest AlCc at all cases). The AICc differences between the “best” and other two candidates were mostly greater than 20. Therefore, we described the decomposition dynamics using double-exponential model parameters (*A*, *k*_1_, *k*_2_). We note that the double-exponential curves best describing decomposition patterns here should be interpreted only within the context of the period of this study. The kinetically defined active pools (A) are 15.5 ± 3.2% in fine roots, and 44.9 ± 6.1% in leaf litter (Table 2).

We used metrics of in situ leaf litter and fine root decomposition to test the effects of simulated N deposition on decomposition. Simulated N deposition did not affect *A* values for leaf litter and fine roots (*F* = 2.61; *P* = 0.205; *F* = 0.23; *P* = 0.640, respectively, Table 2), indicating no change in the size of active pool in both litter types. The effects of simulated N deposition on leaf litter decomposition rates were generally minor (Figure 1; Table 3). Simulated N deposition marginally stimulated the decomposition rates of the active pool (*k*_1_) of leaf litter deployed on O horizon surface (5.15 to 5.74 y^−1^, *F* = 3.46, *P* = 0.081), but there was no effect on the slow pool decomposition rate (*k*_2_, *F* = 0.03, *P* = 0.870, Table 3, 4). Also, there was no effect on the decomposition rate of either pool for leaf litter decomposed at O/A interface (*P* > 0.376, Tables 3, 4). After 3 years of decomposition, simulated N deposition did not significantly alter the final proportion of the remaining leaf litter mass, either on O horizon surface or at O/A interface (*P* > 0.693, Figure 1; Tables 3, 4). When projected to 6 years of decomposition using double-exponential models, the proportions of leaf litter mass remaining, either decomposed on O surface or at O/A interface, were not significantly affected by N additions (*P* > 0.417, Figure 1, Table S1). By contrast, simulated N deposition significantly decreased the decomposition rates of slow pools in fine roots (*k*_2_, 0.175 to 0.144 y^−1^, *F* = 12.3, *P* = 0.003) and increased the final proportion of mass remaining from 51.29 to 56.40% averaged across sites (*F* = 21.2, *P* < 0.001, Figure 1; Tables 3, and 4). However, the increase in final mass remaining was not apparent at site C (site × NO_3_^−^: *F* = 2.72, *P* = 0.079; Figure 1). When projected to 6 years, simulated N deposition increased the proportion of fine root mass remaining from 29.7 to 35.9% (*F* = 15.8, *P* < 0.001, Table S1). To further test whether N additions selectively preserved fine roots over leaf litter, we conducted a two-way ANOVA (Site × NO_3_^−^) on the differences in the proportion of mass remaining between leaf litter and fine roots (Table 5). Simulated N deposition significantly enlarged the difference between proportional mass remaining of leaf litter and fine roots (*F* = 5.14, *P* = 0.037).

The initial chemistry of leaf litter and fine roots varied among sites and as a result of the chronic N amendments (Table 2; full results provided in Xia and others 2015). Here, initial chemical characteristics were used as variables to relate N-induced chemical changes to the variation in decomposition metrics. For leaf litter decomposed on O horizon surface, *k*_1_ values were negatively correlated with soluble phenolics concentrations (ANCOVA, *P* = 0.011) and positively correlated with N concentrations (*P* = 0.036, Table 3). Notably, soluble phenolics and N were not significantly correlated in leaf litter (*P* = 0.348). In contrast, no initial chemical traits had significant correlations with fine root decomposition metrics (ANCOVA, *P* > 0.380).

Fine roots were decomposed reciprocally between ambient and N-added plots to further disentangle the effects of initial substrate and external N availability. Externally supplied N significantly decreased the later-stage decomposition rate (*k*_2_, *F* = 8.21, *P* = 0.011), while substrate source had little effect on *k*_2_ values (*F* = 2.92, *P* = 0.107, Table 6). Moreover, externally supplied N significantly increased the final proportions of root mass remaining after 3 years (F = 15.74, *P* = 0.001, Table 6). Substrate source also altered the proportion of mass remaining, but to a lesser degree (*F* = 5.42, *P* = 0.033). To investigate which biochemical traits were related to the substrate source effects on fine roots decomposition, we conducted an alternative ANCOVA, with site and external N availability as main effects and each of the chemical traits as covariates on decomposition metrics of fine roots. Again, none of the chemical traits had significant effects on these metrics (ANCOVA, *P* > 0.235, Table 6).

At the ecosystem scale, the mass (g m^−2^) of an annual leaf litter cohort remaining after 3 years and that projected for the 6 year was not affected by simulated N deposition (P > 0.385, Table 3, S1). By contrast, N additions marginally increased the quantity of fine root mass remaining after 3 years from 159.7 to 177.8 g m^−2^ (average across sites, *F* = 3.85, *P* = 0.067, Table 3), and significantly increased the projected fine root mass remaining after 6 years by 24.1% (from 91.8 to 113.9 g m^−2^, *F* = 11.7, *P* = 0.003, Table S1). The increase in fine root mass retention was consistently pronounced at site A, B, and D, with the increase after 3 years ranging from 27.3 g m^−2^ at site B to 41.1 gm^−2^ at site A. The increase after 6 years ranged from 29.0 g m^−2^ at site B to 41.5 g m^−2^ at site D. However, this increase did not occur at site C (site × NO_3_^−^: 3 year_mass_, *F* = 3.06, *P* = 0.057; 6 year_mass_, *F* = 4.01, *P* = 0.026), where the averaged quantity of root mass remaining was lower with N additions, but not significantly according to Tukey’s HSD post hoc comparisons at *P* < 0.05.

The site-specific effects of N additions on fine root mass retention were generally consistent with previously documented effects on soil C pools. After 10 years of experimental treatment at MGS sites, the soil C pool in the upper 10 cm of soil under simulated N deposition was observed to be greater than that under ambient conditions (Pregitzer and others 2008). At sites A, B, and D, N additions induced an additional C stock ranging from 273 to 1910 g C m^−2^, with site B showing the largest increase. Site C showed a marginal increase of only 80 g C m^−2^ (Pregitzer and others 2008). Consistent with this, site C did not accumulate more decomposing root mass under simulated N deposition in this study.

To estimate how much greater root mass retention due to simulated N deposition observed in this study could account for soil C accumulation observed in Pregitzer and others (2008), we used a multi-cohort simulation for fine root mass to calculate C retained in the root residues that were continuously added to the soil during 10 years (Method S1). Slower fine root decomposition and thus greater C retained in root residues accounted for 42.9, 4.6, and 50.6% of the documented C accumulation in the surface soil at sites A, B, and D, respectively. We are aware that this estimation is at best an approximation of root-driven C in the soil. This is because other root litter cohorts may have different decomposition dynamics from the one we tracked in this study; also, the C observed to be lost from root residues may still reside in the soil. To address the issue that the C lost from root residues may still reside in the soil, we alternatively estimated the contribution of greater root mass retention to soil C accumulation by including a C partitioning module along with the multi-cohort simulation for root mass. This module partitions C loss from root residues to what was lost from soil as CO_2_ and what remains as microbial biomass and humus. The algorithm of this C partitioning module is based on RothC soil C model (Coleman and Jenkinson 2014, see Method S1 for details). When taking into account the C that was lost from root residues but still remained as microbial biomass and humus in soil, the effect of N additions on fine root decomposition was still estimated to account for 45.8, 4.0, and 52.0% of the documented C accumulation at sites A, B, and D, respectively. These estimations showed that the suppression of elevated N deposition on fine root decomposition observed in this study, if persistent, is biogeochemically meaningful.

## Discussion

We observed that simulated N deposition decreased fine root decomposition rates, but had relatively minor effects on leaf litter decomposition. Excess N has been widely observed to alter litter decomposition (Knorr and others 2005), particularly slowing the breakdown of complex biochemicals such as lignin (Fog 1988; Knorr and others 2005). Previous studies devoted to understand these responses have focused on changes in soil extracellular enzyme activity (for example, DeForest and others 2004; Keeler and others 2009) or changes in soil C pools (for example, Neff and others 2002; Frey and others 2014), including previous research at MGS sites (Pregitzer and others 2008). Studies on the effects of exogenous N on decomposition have primarily focused on leaf litter (Knorr and others 2005; Janssens and others 2010). However, in forests, the annual flux of fine root litter to the soil is broadly similar in magnitude to that of leaf litter and these two litter types represent decomposition substrates that are highly different in initial chemical characteristics such as the concentrations of lignin and condensed tannins (Xia and others 2015). Given that simulated N deposition showed clear effects on later stages of fine root decomposition, but did not affect leaf litter decomposition (Figure 1; Table 3) and has consistently suppressed the activity of peroxidase and phenol oxidase that degrade lignin at these sites (DeForest and others 2004; Edwards and others 2011), it is apparent that large differences between these major litter sources play a large role in the accumulation of soil carbon at these sites.

### Effects of Simulated Nitrogen Deposition on Leaf Litter Decomposition

Simulated N deposition had minor effects on leaf litter decomposition. Nitrogen additions marginally stimulated initial leaf litter decomposition rates (*k*_1_), but had no significant effects on subsequent leaf litter decomposition rates (*k*_2_), the proportion of mass remaining, and the ecosystem-scale estimates of leaf litter mass retention (Figure 1; Table 3). This agrees with other studies showing that experimental N additions often have inconsistent or positive effects on initial decomposition of maple leaf litter and other low-lignin litters (Carreiro and others 2000; Sinsabaugh and others 2002; Knorr and others 2005). In contrast, N additions have been reported to suppress decomposition of high-lignin leaf litters (for example, Sinsabaugh and others 2002; Hobbie and others 2012).

Simulated N deposition stimulated early-stage leaf litter decomposition, an effect that was associated with increased initial (prior to decomposition) leaf litter N concentrations and decreased soluble phenolics concentrations, which occurred as a result of simulated N deposition at four and three of the four study sites, respectively (Table 2, 4). This supports our hypothesis that the effect of simulated N deposition on leaf litter decomposition can be attributed, at least partially, to the way it alters initial leaf litter chemistry prior to decomposition. Although N additions enhanced cellulose-degrading enzyme activity in some ecosystems (Keeler and others 2009; Sun and others 2016), this has not been observed at our sites. Rather, N deposition suppressed *β*-glucosidase activity and did not affect other major cellulolytic enzyme activities (Deforest and others 2004) nor the expression of cellulolytic gene *cbhI* (Edwards and others 2011). This suggests that external N enrichment is less important than initial substrate chemistry for accelerating early-stage leaf litter decomposition at our sites.

High substrate N concentrations have been reported to stimulate early-stage decomposition, yet suppress later degradation (Berg 2000). The increase in leaf litter N concentrations stimulated early-stage decomposition, but did not inhibit later-stage decomposition in this and a similar study (Hobbie and others 2012). A larger range of N concentrations may be needed to reveal the differential effects of substrate N on decomposition (for example, 0.4–3.0% in Berg 2000). The negative effect of soluble phenolics on decomposition is difficult to interpret because phenolics comprise a mixture of different compounds, among which are easily degradable low molecular weight phenolics and more complex compounds that can retard decomposition (for example, condensed tannins, Bhat and others 1998; Hättenschwiler and Jørgensen 2010).

### Effects of Simulated Nitrogen Deposition on Fine Root Decomposition

As expected, the later-stage decomposition rates (*k*_2_) for fine roots were significantly decreased by N additions, leading to an increase in root mass retention at the end of this study (Figure 1; Table 3). The decomposition of leaf litter deployed at the same soil layer as fine roots was not inhibited by N additions (Table 3), indicating that the contrasting responses of leaf litter and fine roots to N additions were due to their intrinsic differences rather than physical environments. The effect of simulated N deposition on later-stage fine root decomposition rate is attributed to the external N enrichment rather than the altered initial substrate quality (Table 6). Consistently, our previous work showed that simulated N deposition had little effect on initial fine root biochemistry (Xia and others 2015)

Although simulated N deposition did not alter the production of fine root litter (Xia and others 2015), slower decomposition with N additions led to greater retention of decomposing fine root mass at three of four MGS sites (Table 3, S1). This greater root mass retention was estimated to explain up to 51% of the previously documented C accumulation in the surface soil with N additions at these sites (Pregitzer and others 2008). On the other hand, the fact that a significant proportion of soil C accumulation cannot be explained by slower mass loss of fine roots implies that other mechanisms also contribute to soil C accumulation under simulated N deposition. A recent study at our sites found that N additions increased mineral-occluded particulate organic matter (Zak and others 2017), suggesting greater mineral affiliation is also a driver for soil C accumulation with elevated N deposition. No additional retention of decomposing root mass was observed at site C, which is partly due to lower fine root litter flux under simulated N deposition at this site (Xia and others 2015). Consistent with this, the increase in soil C with N additions at site C was the lowest of all sites in our previous analysis (Pregitzer and others 2008).

High levels of exogenous N have been observed to suppress lignin degradation, which dominates later stages (>1 y) of litter decomposition (Berg 2000). Under laboratory conditions, high N availability repressed metabolic activities associated with lignin degradation in *Phanerochaete chrysosporium* (Boominathan and others 1990). Field studies in temperate/boreal forests have found that N fertilization inhibited lignin degradation (Berg and Tamm 1991; Magill and Aber 1998; but Hobbie 2008). Previous work at MGS sites observed that simulated N deposition suppressed the activity of lignin-degrading extracellular phenol oxidase and peroxidase (DeForest and others 2004) and down-regulated expression of the *Icc* gene encoding for laccase (Edward and others 2011). Recently, Freedman and others (2016) reported that simulated N deposition shifted saprotrophic microbial community toward bacterial metabolisms that are less oxidatively powerful compared to fungal pathways in lignin degradation. These results, along with the observation that lignin was a major fraction in fine root tissue at MGS sites (Xia and others 2015), support the idea that simulated N deposition slows later-stage decomposition of fine roots as a result of depressed lignin degradation metabolism. Because fine roots are generally lignin-rich materials in temperate and boreal forests (Rasse and others 2005; Xia and others 2015), our results suggest that decomposing fine roots could represent a growing C sink in these forests as high rates of anthropogenic N deposition persist in North America and Europe, as well as become more widespread in developing regions.

### Mass Loss and Model Projection

The double-exponential model described mass loss over time well in both leaf litter and fine roots. Multi-pool models have been reported to perform “better” than single-phase models in fitting litter decomposition, with double-exponential models (for example, Harmon and others 2009; Alexander and Arthur 2014) or asymptotic models (for example, Hobbie and others 2012; Sun and others 2016) often best describing decomposition data. These multi-pool models are consistent with the idea that compounds such as sugars, starch, and simple phenolics form a labile pool that leaches/decomposes quickly, with subsequent decomposition limited by more recalcitrant polymers such as lignocellulose, condensed tannins, and cutin (Berg 2000; Preston and others 2009). Single decay models assume a constant decomposition rate for litter material and has been observed to underestimate the mass remaining toward the later stages of decomposition (Manzoni and others 2012), as was the case in this study (data not shown).

We compared the decomposition dynamics of leaf litter decomposed on O horizon surface to a long-term multi-site decomposition experiment database (LIDET, Harmon 2013) where leaf litter was also deployed on the ground surface. The proportion of mass remaining at the end of this 3-year study (averaged across sites, 25.0%) and the model-projected proportion after 6 years (12.1%) were comparable to that measured within LIDET at three other cool temperate broadleaf forests in the eastern USA (Coweeta Hydrologic Laboratory, Hubbard Brook Experimental Forest, and Harvard Forest). Sugar maple leaf litter at those three forests displayed an average 28.5% mass remaining (from 19.5 to 41.1%) at the third year, and 15.4% (7.1–27.9%) at the sixth year. However, compared to our projections, sugar maple leaf litter showed a slower decomposition at a temperate-mixed conifer-hardwood forest (North Temperate Lakes site within LIDET) and a northern hardwood forest that is cooler than three of the four MGS sites (Lovett and others 2016).

For fine roots, the mass remaining estimated in our study seems somewhat higher than that for the broadleaf tree roots tracked in LIDET: 41.2 and 25.6% of *Drypetes glauca* fine root mass remained across those three temperate forests in the third and sixth year. These roots had a much lower AIF concentration (16.3%) than the sugar maple roots in this study (Table 2). Also, LIDET used fine roots with a diameter below 2 mm, whereas we used the distal three order roots, mostly less than 0.5 mm in diameter. The distal small-diameter roots generally decompose slower than larger roots, probably due to lower C/N ratios, higher contents of AIF, and the presence of mycorrhizal colonization (Fan and Guo 2010; Goebel and others 2011). Using roots less than 0.5 mm, Sun and others (2016) reported approximately 40% of root mass remaining after 5 years averaged across five temperate broadleaf litters, which is similar to the model projection in this study (38.3% after 5 years, data not shown).

In summary, we observed that simulated N deposition had minor effects on leaf litter decomposition, but significantly inhibited the later stages of fine root decomposition. This inhibition is likely caused by the decreases in lignin-degrading enzyme activities. At the ecosystem scale, slower fine root decomposition led to greater root mass retention and was estimated to explain 5–51% of previously documented increase in surface soil C under simulated N deposition. Although previous N deposition studies have focused on leaf litter decomposition, our study showed that fine roots are an important driver to the soil organic carbon accumulation under simulated N deposition. Most earth system models do not include N interactions with soil C, with a few using N limitations on decomposition alone to represent C–N coupling (Todd-Brown and others 2013). This and previous studies on these long-term N manipulated sites suggest the need for a more comprehensive N and C interaction framework that takes into account the possible suppression of litter decomposition and additional soil carbon sequestration in the scenario of increasing N deposition.

## Supplementary Material

Supplement1

## Figures and Tables

**Table 1.** Site Characteristics of Four Northern Hardwood Forests

Site characteristic	Site A	Site B	Site C	Site D
Latitude (N)	46°52′	45°33′	44°23′	43°40′
Longitude (W)	88°53′	84°51′	85°50′	86°09′
Mean annual precipitation (mm)^a^	873	871	888	812
Growing season precipitation (mm)^b^	401	388	393	379
Mean annual temperature (°C)^a^	4.7	6.0	6.9	7.6
Growing season temperature (°C)^b^	15.0	16.0	16.2	16.8
Total basal area (m^2^ ha^−1^)^c^	34	31	32	33
Sugar maple basal area (%)^c^	86	86	83	75
Ambient wet + dry N deposition (g N m^−2^ y^−1^)^d^	0.68	0.91	1.17	1.18
Soil texture, 0–10 cm depth (%sand–%silt–%clay)^a^	75–22–3	89–9–2	89–9–2	87–10–3
Soil texture, 10–70 cm depth (%sand–%silt–%clay)^a^	84–11–5	88–7–5	91–6–3	92–5–3

a Pregitzer and others (2008);

b Burton and others (2012);

c Burton and others (2000);

d MacDonald and others (1992).

**Table 2.** Chemical Characteristics (%) and Kinetically Determined Active Pool (*A*, %) of Initial Litter Collected from Ambient and NO_3_^−^ Amended Plots

Site	Litter type	Litter source	EXT	AIF	ASF	PHE	CTs	Lipids	NSCs	PRO	N	C/N	AIF/N	LCI	*A*
A	Leaf litter	Ambient	39.30	16.18	44.52	13.61	9.08	8.50	5.26	2.02	0.79	63.33	20.38	0.27	51.7
		NO_3_^−^	43.45	14.66	41.89	15.09	6.92	8.28	7.14	1.48	0.83	61.32	17.74	0.26	50.7
	Fine roots	Ambient	17.51 (1.18)	42.71 (1.09)	39.79 (1.07)	3.88 (0.21)	14.83 (0.77)	3.72 (0.29)	2.09 (0.08)	3.20 (0.07)	1.35 (0.02)	37.38 (0.59)	31.73 (0.68)	0.52 (0.01)	15.9 (2.0)
		NO_3_^−^	18.09 (0.51)	45.49 (1.76)	36.43 (1.64)	4.03 (0.11)	15.82 (1.13)	3.54 (0.33)	2.10 (0.32)	3.30 (0.26)	1.55 (0.06)	33.66 (1.43)	29.42 (1.44)	0.56 (0.02)	15.3 (1.7)
B	Leaf litter	Ambient	37.00	15.05	47.95	13.53	5.22	7.67	4.98	0.78	0.66	71.22	22.65	0.24	49.4
		NO_3_^−^	39.33	14.08	46.59	11.60	3.54	6.98	6.10	0.73	0.97	50.27	14.46	0.23	47.4
	Fine roots	Ambient	15.68 (0.50)	45.34 (0.49)	38.98 (3.14)	3.24 (0.24)	11.70 (1.25)	3.72 (0.21)	1.73 (0.24)	3.24 (0.25)	1.74 (0.11)	29.05 (1.80)	26.30 (1.92)	0.54 (0.01)	19.0 (2.8)
		NO_3_^−^	13.96 (0.22)	45.72 (0.81)	40.32 (2.75)	2.99 (0.34)	10.49 (0.50)	3.18 (0.43)	1.71 (0.10)	2.61 (0.28)	1.72 (0.06)	29.32 (1.02)	26.63 (1.31)	0.53 (0.01)	16.4 (0.8)
C	Leaf litter	Ambient	38.84	14.44	46.73	13.29	6.11	7.64	5.88	1.20	0.64	77.39	22.60	0.24	46.0
		NO_3_^−^	39.07	14.22	46.71	10.60	4.19	6.88	4.91	1.25	0.83	58.33	17.08	0.23	43.2
	Fine roots	Ambient	15.90 (1.71)	45.90 (1.37)	38.20 (1.62)	4.11 (0.73)	13.02 (2.54)	3.07 (0.56)	1.74 (0.07)	3.58 (0.72)	1.66 (0.05)	31.33 (1.01)	27.84 (1.61)	0.55 (0.01)	15.9 (5.1)
		NO_3_^−^	13.76 (1.11)	46.72 (0.72)	39.62 (4.32)	3.16 (0.28)	9.90 (1.50)	3.31 (0.32)	1.66 (0.29)	2.80 (0.28)	1.78 (0.04)	29.06 (0.63)	26.36 (0.27)	0.54 (0.01)	16.3 (5.5)
D	Leaf litter	Ambient	39.51	14.74	45.75	11.82	4.48	8.17	4.76	0.91	0.73	68.68	20.29	0.24	35.2
		NO_3_^−^	36.72	14.67	48.60	10.69	5.08	8.91	5.53	1.34	0.85	58.11	17.22	0.23	36.0
	Fine roots	Ambient	16.58 (0.90)	46.42 (2.36)	37.00 (4.96)	4.57 (0.47)	14.82 (1.02)	3.38 (0.27)	1.94 (0.23)	3.12 (0.34)	1.46 (0.15)	35.75 (3.99)	32.32 (4.78)	0.56 (0.03)	12.8 (1.0)
		NO_3_^−^	16.68 (2.35)	45.16 (1.27)	38.17 (1.92)	4.79 (0.96)	13.36 (2.42)	3.60 (0.07)	1.92 (0.52)	3.08 (0.39)	1.51 (0.12)	34.59 (2.98)	30.19 (3.20)	0.54 (0.01)	13.1 (1.3)

Values of leaf litter are homogenized leaf litter combined from three ambient or NO_3_^−^ amended plots at each site. Values of fine roots are means (SD) for three ambient or NO_3_^−^ amended plots (n = 3), which have been documented in Xia and others (2015).

EXT, extractive fraction; AIF, acid-insoluble fraction; ASF, acid-soluble fraction; PHE, soluble phenolics; CTs, condensed tannins; NSCs, nonstructural carbohydrates; PRO, soluble proteins; N, nitrogen; LCI, lignocellulose index, AIF/(AIF + ASF).

**Table 3.** Decomposition Rates (*k*_1_, *k*_2_), Proportions of Mass Remaining (3 year_percentage_, %), and Quantities of Mass Remaining (3 year_mass_, g m^−2^) of an Annual Litter Cohort for Leaf Litter and Fine Roots Decomposed in situ for 3 Years Across Four Northern Hardwood Forests

Decomposition metrics	Leaf litter (O/A interface)		Leaf litter (O surface)		Fine roots	
	Ambient	NO_3_^−^	Ambient	NO_3_^−^	Ambient	NO_3_^−^
*k*_1_ (y^−1^)	11.02 (2.46)	11.52 (2.50)	5.15 (0.69)	5.74(*) (1.22)	16.97 (10.70)	19.30 (7.55)
*k*_2_ (y^−1^)	0.504 (0.104)	0.513 (0.087)	0.266 (0.078)	0.264 (0.096)	0.175 (0.024)	0.144** (0.018)
3 year_percentage_ (%)	14.96 (4.15)	15.06 (3.31)	24.60 (3.46)	25.41 (5.19)	51.29 (2.49)	56.40***^, ‡^ (3.23)
3 year_mass_ (g m^−2^)			91.97 (15.62)	97.47 (19.18)	159.70 (33.63)	177.84(*)^, ‡^ (60.97)

Values are means (SD) of decomposition indices of leaf litter and fine roots across plots in either ambient conditions or NO_3_^−^ treatment (n = 12).

(*), **, *** Significant effects of simulated N deposition at P < 0.10, P < 0.01. P < 0.001, respectively. Only leaf litter decomposed at O horizon surface was used to construct the quantity of mass remaining.

‡ Simulated N deposition increased 3 year_percentage_ and 3 year_mass_ for fine roots at all sites except site C, leading to a significant site × NO_3_^−^ interaction (P < 0.079, Table 4; Figure 1).

**Table 4.** Analysis of Variance for the Effects of Site (*df* = 3), Simulated N Deposition (*df* = 1), and Their Interaction (*df* = 3) on Decomposition Rates (*k*_1_, *k*_2_), Proportions of Mass Remaining (3 year_percentage_) and Mass Remaining Per Area Unit (3 year_mass_) After 3 Years for Leaf Litter and Fine Roots Decomposed in situ Across Four Northern Hardwood Forests

Source of variance	*k*_1_		*k*_2_		3 year_perrcentage_		3 year_mass_	
	*F*	*P*	*F*	*P*	*F*	*P*	*F*	*P*
Leaf litter (O/A interface)								
Site	19.3	<**0.001**	16.3	<**0.001**	2.11	0.140		
NO_3_^−^	0.83	0.376	0.21	0.653	0.01	0.932		
Site × NO_3_^−^	1.15	0.358	2.46	0.101	1.57	0.235		
Covariates^a^	ns		ns		ns			
Leaf litter (O surface)								
Site	10.8	**0.001**	17.9	**<0.001**	1.76	0.194	0.14	0.935
NO_3_^−^	3.46	0.081	0.03	0.870	0.16	0.693	0.46	0.507
Site × NO_3_^−^	1.39	0.281	0.59	0.632	0.48	0.703	0.22	0.880
Covariates^a^	PHE^(−)^, N^(+)^		ns		ns		ns	
Fine roots								
Site	2.96	0.063	0.24	0.869	0.22	0.882	26.3	<**0.001**
NO_3_^−^	1.07	0.317	12.3	**0.003**	21.2	<**0.001**	3.85	0.067
Site × NO_3_^−^	0.19	0.903	1.56	0.238	2.72	0.079	3.06	0.057
Covariates^a^	ns		ns		ns		ns	

Statistical results with P values <0.05 are in bold. To estimate the leaf litter mass remaining per unit area, annual leaf litter production data were combined with the decomposition rates of leaf litter only at O horizon surface. Therefore, there are no values for leaf litter 3 year_mass_ at O/A interface.

PHE, soluble phenolics; N, nitrogen.

a The significance of covariates is tested in an alternative ANCOVA with site as the main effect to determine if variation in decomposition indices can be linked to the differential responses of initial chemical characteristics to simulated N deposition. Covariates with P values <0.05 are listed in the table, followed by “^(−)^” or “^(+)^” to indicate a negative or positive correlation, with “ns” denoting no significant covariates.

**Table 5.** Analysis of Variance on the Differences Between Percentages of Mass Remaining of Fine Roots and Leaf Litter (% M_R–L_) Among Sites (*df* = 3) and Simulated N Deposition (*df* = 1)

Effects	n/m	*F*	*P*
Site	3/16	1.71	0.204
NO_3_^−^	1/16	5.14	**0.037**
Site × NO_3_^−^	3/16	2.29	0.118

Bold value indicates statistical significance (p < 0.05)

The values of % M_R–L_ are computed as the percentage mass remaining of fine roots after 3 years minus that of leaf litter (O horizon surface) in each plot. We used “% M_R–L_” to test if experimental NO_3_^−^ amendment selectively reserve fine root mass over leaf litter. The degrees of freedom, numerator df/denominator df, are shown as “n/m.”

**Table 6.** Mixed Linear Model Analysis on a Split-plot Design Testing the Differences of Fine Root Decomposition Rates (*k*_1_, *k*_2_) and Percentages of Mass Remaining After 3 Years (3y %) Among Study Sites, External N Availability, and Substrate Source (Fine Roots Collected From Ambient vs. NO_3_^−^ Amended Plots)

Source of variance	n/m	*k*_1_		*k*_2_		3y %	
		*F*	*P*	*F*	*P*	*F*	*P*
Site	3/16	3.74	**0.033**	0.17	0.918	1.80	0.188
External N availability	1/16	0.97	0.340	8.21	**0.011**	15.74	**0.001**
Site × N	3/16	0.26	0.852	0.80	0.512	1.14	0.362
Substrate source	1/16	0.16	0.695	2.92	0.107	5.42	**0.033**
Site × source	3/16	1.61	0.226	1.33	0.298	2.67	0.083
N × source	1/16	1.01	0.329	0.03	0.860	2.07	0.169
Site × N × source	3/16	0.31	0.815	1.01	0.413	1.86	0.178
Covariates^a^		ns		ns		ns	

Bold value indicates statistical significance (p < 0.05)

The degrees of freedom, numerator df/denominator df, are shown as “n/m.”

a The significance of covariates is tested in an alternative ANCOVA with site and external N availability as main effects and each of initial chemical traits as a covariate. “ns” means no significant covariates (at P < 0.05) were found.
