# Supplementary material for: Long-Term Simulated Atmospheric Nitrogen Deposition Alters Leaf and Fine Root Decomposition
Source: Ecosystems. Author manuscript; Available in PMC 2019 May 29. (PMC6541405; doi:10.1007/s10021-017-0130-3)
Supplement: Supplement1 [file NIHMS1504309-supplement-Supplement1.docx]

**Electronic Supplementary Files**

**Table S1** The Model-projected Proportions of Mass Remaining (6y_percentage_, %) and Quantities of Mass Remaining (6y_mass_, g m^-2^) of an Annual Litter Cohort for Leaf Litter and Fine Roots Decomposed *in situ* for Six Years Across Four Northern Hardwood Forests

| Decomposition metrics | | Leaf litter  (O/A interface) | | | Leaf litter  (O surface) | | | Fine roots | |
| --- | --- | --- | --- | --- | --- | --- | --- | --- | --- |
|  |  | Ambient | NO_3_^-^ |  | Ambient | NO_3_^-^ |  | Ambient | NO_3_^-^ |
| 6y_percentage_ (%) |  | 2.95  (1.38) | 2.82  (1.36) | | 11.64  (4.11) | 12.65  (5.83) | | 29.68  (3.86) | 35.94***  (4.12) |
| 6y_mass_ (g m^-2^) |  |  |  | | 42.69  (12.21) | 47.25  (17.93) | | 91.76  (18.92) | 113.89**^‡^  (40.76) |

Values are means (SD) of decomposition indices for leaf litter or fine roots across plots in either ambient conditions or the NO_3_^-^ treatment (n = 12). The projected proportion of mass remaining was predicted by double exponential decay models in each plot. The quantity of mass remaining of annual litter input as a cohort over time was estimated by multiplying litter input rates (see Xia and others 2015) with the projected proportion of mass remaining in each plot. **, *** denote significant effects of simulated N deposition at *P* < 0.01 and *P* < 0.001, respectively. Only leaf litter deployed on O horizon surface was used to construct the quantity of mass remaining. ^‡^Simulated N deposition significantly increased 6yr_mass_ for fine roots at all sites except site C, leading to a significant site × NO_3_^-^ interaction (*P* = 0.026, data not shown). We note that the statistical analysis was performed on projected data with an assumption that the projected values represent the realization of the corresponding random variables. The statistical inferences based on this assumption should be interpreted with caution.

**Methods S1** Multi-cohort simulation of root-driven carbon in the soil

Carbon (C) in the root residues that were continuously added to soil (*SOC*_R_) was calculated as

$${SOC}_{R}=\sum_{j=0}^{n} P\cdot M_{C}(j)$$

In this formula, *P* indicates fine root litter input (g m^-2^) in the upper 10 cm soil on a plot basis (see Xia and others 2015 for the estimation of root litter input data at the plot level); *j* is the age (year) of a certain litter cohort. We aimed to estimate how much slower root decomposition can account for the C accumulation in the upper 10 cm soil that was reported in Pregitzer and others (2008). We set *n* to 10 because Pregitzer and others (2008) measured soil C in 2004, ten years after the beginning of the treatment at our sites since 1994. *Mc(j)* is the double exponential decomposition function multiplied with carbon concentrations to represents the percentage of carbon remaining for a certain litter cohort at the age of *j*. The double exponential decomposition function was parameterized in this study on the basis of plot-level data.

Alternatively, we added a C partitioning module to the above simulation of *SOC*_R_ to take into account the C that was lost from root residues but still remains as microbial biomass and humus in soil:

$${{SOC}_{T}=SOC}_{R}+ {SOC}_{BH}$$

$${SOC}_{BH}=\sum_{j=1}^{n} \sum_{i=1}^{j} (P\cdot(M_{C}\left( i-1 \right)-M_{C}\left( i \right))\cdot(BIO\cdot e^{-k_{B}\cdot\left( j-i \right)}+HUM\cdot e^{-k_{H}\cdot\left( j-i \right)})$$

where *SOC*_BH_ represents the C that was lost from root residues but remains in the soil as microbial biomass and humus, *SOC*_T_ represent the total root-driven C originated from root residues continuously added to soil, and *i* is the age of the litter when the microbial biomass (BIO) and humus (HUM) were derived. The algorithm of C partitioning is based on that of RothC 26.3 model (Coleman and Jenkinson 2014). Specifically, the proportion of the C loss that goes to BIO and HUM is determined by the clay content of the soil as:

$$BIO+HUM =\frac{1}{1.67(1.85+1.60\exp\left( -0.0786\%clay \right)+1}$$

The BIO+HUM is then partitioned into 46% BIO and 54% HUM. The generated microbial biomass and humus further degrade at a rate of *k*_B_ and *k*_H_ respectively, which are functions of air temperature, soil moisture deficit, and soil cover modifying factor (see Coleman & Jenkinson 2014 for details). Soil texture data, air temperature, and soil water balance for our sites that are needed to estimate *k*_B_ and *k*_H_ are available from the Michigan Nitrogen Deposition Gradient Study database, <http://webpages.uidaho.edu/nitrogen-gradient>. The calculation of *SOC*_BH_ includes microbial biomass C and humus-C currently partitioned from recent C loss for a litter cohort at the age of *j* (when *j* = *i*), and also includes microbial biomass C and humus-C partitioned from C loss during each of the previous years (when *j* $>$ *i*) which then decayed at rates of *k_B_* and *k_H_* respectively for a time period of (*j*-*i*). The percent contributions of the slower root decomposition to the soil C accumulation under experimental nitrogen deposition were estimated as the average difference in *SOC*_R_ or *SOC*_T_ between ambient and nitrogen-amended treatments divided by the documented soil C increase at each site.

**Reference**

Pregitzer KS, Burton AJ, Zak DR, Talhelm AF. 2008. Simulated chronic N deposition increases carbon storage in northern temperate forests. Glob Change Biol 14: 142–153.

Coleman K, Jenkinson DS. 2014. RothC - a model for the turnover of carbon in soil. Model description and users guide. Rothamsted Research, Harpenden, UK. Available at: http://rothamsted.ac.uk/sites/default/files/users/kcoleman/RothC_guide_DOS.pdf.

Xia M, Talhelm AF, Pregitzer KS. 2015. Fine roots are the dominant source of recalcitrant plant litter in sugar maple‐dominated northern hardwood forests. New Phytol 208:715-726.
